# Assessment of liver function test and associated factors among visceral leishmaniasis patients attending university of gondar leishmaniasis research and treatment center, Northwest Ethiopia

**DOI:** 10.1371/journal.pone.0260022

**Published:** 2021-11-19

**Authors:** Hiwot Tezera Endale, Tiget Ayelgn Mengstie, Dilargachew Dessie Dawit, Rezika Mohammed, Gashaw Dessie, Kibur Hunie Tesfa

**Affiliations:** 1 Department of Medical Biochemistry, School of Medicine, College of Medicine and Health Sciences, University of Gondar, Gondar, Ethiopia; 2 Leishmaniasis Treatment and Research Center of University of Gondar Comprehensive Specialized Hospital, Gondar, Ethiopia; Oregon State University, UNITED STATES

## Abstract

**Background:**

Visceral leishmaniasis (VL) is one of the major public health burden, mainly distributed throughout tropical and subtropical regions of the world. Among the Sub-Saharan African countries, Ethiopia is the second most affected country with VL. An Alteration of liver function is a typical manifestation of the disease.

**Objective:**

The purpose of conducting this study was to assess liver function tests and associated risk factors among VL patients at Leishmaniasis Research and Treatment Center of University of Gondar Comprehensive Specialized Hospital, North West Ethiopia.

**Method:**

Hospital based comparative cross-sectional study design was conducted. A total of 102 study participants were involved in this study. Newly diagnosed VL patients who were attended at Leishmaniasis Research and Treatment Center of University of Gondar Comprehensive Specialized Hospital from 21^st^ February 2020 to 30th September 2020 were included under case group category. On the other hand, age-sex matched apparently healthy study subjects were categorized as control group. Written consent was obtained willingness of patients to participate after ethical clearance was obtained from the Institutional Review Board of School of Medicine, University of Gondar. After overnight fasting, 5ml venous blood was drawn from both VL patients and controls to evaluate liver function tests, including AST, ALT, total bilirubin, albumin, and total protein. Thus, senior health professionals (laboratory technologist) investigate the results using Cobas Integra 400 Plus clinical chemistry analyzer. Data was entered into Epi-data version 4.6 and exported to STATA 14 for analysis of liver function tests and associated risk factors.

**Result:**

The result of this study showed that significant mean difference was exhibited in aspartate aminotransferase (AST), alanine aminotransferase (ALT), total bilirubin, serum albumin, and total protein level among VL patients and controls. It showed that there was a statistically significant elevation in the level of AST, ALT, and total bilirubin among cases as compared to control. The serum AST level was significantly (p<0.001) elevated among cases as compared to controls. Serum ALT was significantly (p<0.001) elevated among cases compared to controls. Additionally, the total serum bilirubin level was significantly increased (P<0.001) among cases as compared to controls. There was a statistically significant (P<0.001) reduction of serum albumin level among VL patients as compared to controls. Similarly, serum total protein was significantly (P<0.001) reduced in VL patients than control groups.

**Conclusion:**

There were significantly higher mean levels of serum AST, ALT, and total bilirubin among VL patients as compared to controls. On the other hand, VL patients showed significantly lowered level of albumin and total protein as compared to controls.

## Introduction

Leishmaniasis is a tropical disorder caused by pathogenic genus family leshimania, and it infects over 12 million population worldwide [1]. Globally, it is the major public health burden, which is commonly disseminated in the area of subtropical and tropical regions [2]. Leishmaniasis is commonly distributed in 88 countries worldwide. Regard to its endemic, 2 million people are newly infected with this disease annually (1.5 million cutaneous leishmaniasis and 0.5 million people with visceral leshimaniasis) [3]. The incidence of VL expected to be 50,000 to 90,000 new cases every year in the world. It is mainly disseminated in the region of Mediterranean, South America, South Asia, and East Africa [4, 5]. Even though the emphasis given to the disease is low, the mortality rate of the disease is increased in every continent except Australia and Antarctica [5]. The magnitude of the problem and newly infected cases is associated with truck driver, sex workers, refugees, seasonal workers, and migrants in India and East Africa region [6]. In Subtropical and tropical area of globe, the infection of VL is endemic [3].

The transmission of the disease is due to a bite of parasite infected female phlebotomine sandflies. Over 20 leshimania species are identified as a causative factor for the pathogenesis of the disease. It has three disease patterns, including visceral, cutaneous, and mucocutaneous leishmaniasis [5]. Visceral leishmaniasis (VL) is the most severe and fatal type of leshimaniasis. *Leishmania donovani* complex is the causative pathogenic parasite for the infection and pathogenesis of VL. *Leshimania infantum* and *donovani* are the most common related species of the complex parasite [5]. In 1930, William leishman and Charles Donovani discover the parasitic species leshimania donovani through spleen biopsy and smear examination [6]. As the parasite evades the host cell, it activates humoral, cellular immunological response, and induction of B-cell activation. Thus, the abnormal immune response in turn leads to damage of tissues, including liver, kidney, spleen, bone marrow, and others [7]. An abnormality in disease activity and immune response in VL is related with malnutrition status of patients [8]. The macrophage found in liver, lymph nodes, kidney, and other tissues evaded with pathogenic parasite. As a result, the macrophages also release unstable free radicals, which leads to damage of lipids, protein, and DNA [9]. The amastigote stage of pathogenic parasite undergo proliferation in liver, bone marrow, and spleen mononuclear phagocytic system (MPS), which leads to an abnormality in those active organ [10]. Studies done on histopathological examination of liver in VL patients showed the presence of hyperplasia of kupffer cells, necrosis, and amastigotes. In addition to this, fibrosis also reported during histopathological examination. Thus, liver damage may occur due to the effect of parasitic invasion of macrophage and kupffer cells [11, 12]. On the other hand, the host cell immune system dysfunction can be developed by altering cell-mediated immune system [10, 12]. Parasitic-associated immunological disturbance leads to the imbalance between pro-inflammatory and anti-inflammatory cytokines. An increase in pro-inflammatory cytokines and a decreased in anti-inflammatory cytokines induce damage to the tissue [13].

The prevalence of VL in East Africa is estimated to be the highest one next to India. Ethiopia is one of highly affected country with VL among sub-Saharan African countries. In 1942, in the area of south west part of the Ethiopia (in lower omo plains), the first VL cases was identified [14]. The morbidity and mortality rate VL increased in highly prevalent area of the country. The country with low socio-economic status, including Ethiopia is mainly affected by the disease. In Ethiopia, 4000 newly infected patients are reported every year. In addition, 3.2 million people are expected to have a risk of VL in the country. The magnitude of problem is high in Northwest part of the country, which accounts for 60% of VL cases [15, 16]. The majority of local regional state, such as Benshangul Gumuz, Gambela, Somali, Southern Nations and Nationalities People’s Region (SNNPR), Oromia, Amhara, and Tigray are highly prevalent area with the disease. The semi-arid and arid area of endemic regions (accounts for 4000 new cases every year) are mostly affected by the disease [17, 18]. The specific northwest part of country, including Fogera (libokemkem), Shiraro, Wolkait, humera, and Metema area are mostly affected by VL [15, 19].

Investigation done in different countries showed that evaluation of nutritional status and liver function tests have a prognostic, therapy management, and treatment significance in VL patients. However, the majority of those studies were done on animal models. There is no adequate comprehensive research done in Ethiopia specific to the study area. Therefore, this study was primarily focused to evaluate liver function tests (AST, ALT, total bilirubin, albumin, and total protein) and factors associated with liver function test among confirmed visceral leishmaniasis patients and apparently healthy individuals at Gondar University Hospital, Northwest, Ethiopia. It will act as a baseline for health professionals to consider these tests during the diagnosis and management of visceral leishmaniasis patients. Additionally, this study supports policy-makers to give emphasis on those laboratory tests to manage the further complication of the disease.

## Methods and materials

### Study area and period

Study was conducted at Leishmaniasis Research and Treatment Center of University of Gondar Comprehensive Specialized Hospital, from February to September 2020. It is located in the Gondar town under the Central Gondar Zone of Amhara Regional state in 12.6 Latitude and 37.47 Longitude. It is found at elevation 2210 meters above sea level and 738 Km far from the capital Addis Ababa. University of Gondar Comprehensive Specialized Hospital is a referral hospital with 500 beds, serving approximately 5 million people and plays an important role in teaching learning process for medical field students over 60 years. In 2004, Leishmaniasis Research and Treatment Center with 24 beds capacity and organized laboratory diagnosis was built up to reduce the disease burden.

### Study design

Hospital based comparative cross-sectional study design was conducted to assess liver function test and associated factors among patients with VL and apparently healthy controls. It was conducted at University of Gondar Comprehensive Specialized Hospital leishmaniasis Research and Treatment Center from 21^st^ February 2020 to 30th September 2020.

### Source and study population

In this study, all lesishmaniasis patients who attended at Leishmaniasis Research and Treatment Center of University of Gondar Comprehensive Specialized Hospital were served as a source population. All newly diagnosed VL patients who attended at research and treatment center during study period were categorized under case groups. In the same environmental setting, apparently healthy individuals who had no chronic complications and willingness to provide signed informed consent were selected for control groups. In addition to this, age-sex matched to the patient was considered during selection of control groups.

### Eligibility criteria of study participants

All newly diagnosed VL patients who were attended at University of Gondar Comprehensive Specialized Hospital and those patients above the age of 18 years old were included in this study. On the other hand, patients with chronic diseases, such as diabetes mellitus, hypertension, liver disease, HIV/AIDS, kidney disease, medication and organ transplant, smoking, and alcohol history were excluded from this study during the study period. On the other hand, the apparently healthy individuals who had no chronic diseases, such as, diabetes mellitus, tuberculosis, hypertension, kidney disease, rheumatoid arthritis, and other disorders were included as control groups during this study. In addition to this, individuals with mental health problems, hearing impairment, any other serious health problems or chronic disease like cancer, history of smoking, and alcohol habit and those who were not able to provide the appropriate information were excluded from control groups. Individuals who were volunteers, 18 years old, and above were included as control group.

### Blood sample collection and analysis process

After overnight fasting, 5ml venous blood was drawn from study participants by BD Vacutainer containing EDTA tube to measure the level of liver function tests. Then, the sample was centrifuged at 3500 revolutions per minute for 5 minutes to separate serum from whole blood components. The sample was preserved at −20°C refrigeration until the biochemical measurements analyzed. The liver function tests were measured using fully automated mind array, calibrated, and Cobas Integra 400 Plus (Roche Diagnostics GmbH, Mannheim, Germany) clinical chemistry analyzer following the instruction of manufacturer. The sample was preserved at −80°C for further visceral leshimaniasis investigation up to six months or beyond this time as necessarily.

### Sample size determination and sampling technique

To determine sample size, the study used GPowerVersion3.1.9.2 as a tool. GPower is one of the software packages that perform sample size calculations like Minitab and Epi-info covering a wider range of study designs. As an input GPower requires selecting appropriate test family, statistical test with in test family, specifying α error probability, power (1-β error probability) and effect size [20]. By considering alpha = 0.05, power (1- β) = 0.8 (80%) and effect size = 0.5 the total sample size became 102 and simple random sampling technique were used.

### Study variables

In this study, the dependent variables were AST, ALT, total bilirubin, serum albumin, and total serum protein, whereas the independent variables included socio-demographic factors, such as age, sex, BMI, and VL disease status.

#### Operational definitions

Abnormal liver function test is identified as more than one of these tests, including AST, ALT, bilirubin, and albumin showed an abnormal finding [21]. The normal value of liver function tests, including AST (1-37U/L), ALT (1-42U/L), total bilirubin (0–1.2 mg/dL), albumin (3.8–5.1g/dL), and total protein(6.6–8.7 g/dL) are falls within those ranges during analysis of result of study participants [22, 23]. On the other hand, anthropometric indicators are parameters for the measurement of the human body and its individual parts. Thus, it yields a quantitative index of their variability. The height, weight, and body mass index are some of the anthropometric measurement parameters [24].

### Data processing and analysis

Data were cleaned, coded, and entered into Epi Data version 4.6 software, and exported to STATA version 14.1 for further analysis of liver function tests and risk factors. The biochemical parameters and socio-demographic characteristics of study participants were analyzed using simple descriptive statistics. In addition, the data was presented using tables, figures, and charts for clarification of the study. The socio-demographic and clinical characteristics were compared among case and control groups using independent t-test. Socio-demographic characteristics and biochemical parameters were compared between groups and within groups using one way ANOVA statistical analysis method. All variables with a p-value of less than 0.2 during binary linear regression analysis were selected for multivariable linear regression analysis.

### Ethical consideration

Ethical approval was obtained from the Institutional Review Board of School of Medicine, College of Medicine and Health Science, University of Gondar. Department of ethics and research committee with the reference number 1998/03/2020 approved it.

## Result

### Socio-demographic characteristics of the study participants

A total of 102 subjects were involved in this study. Among the total study participants, 51 of them were visceral leshimaniasis (VL) patients and 51 of them were apparently healthy controls. All the study participants were male. The Mean ± SD of ages among VL patients and controls were 24± 5.77 and 28.98 ± 6.43 respectively. Patients and controls had a minimum age of 18 and 19 years and a maximum age of 41 and 43 years respectively. The control groups were volunteers and apparently healthy individuals. Majority of VL patients (54.9%) were found within the age group of 18–24 years. In this study, 78.43% of VL patients were single, rural residents, and illiterate. 96.1% and 94.1% of the patients were Orthodox Christian and laborers respectively. 50.9% of the control group falls within the age group of 24–34 years and 70% of them completed secondary education and above. On the other hand, 24(47%), 40(78.4%), 19 (37.3%) and 28 (54.9%) of controls were married, living in urban area, government employees, and Orthodox Christian respectively. Socio-demographic characteristic of the case and control groups are depicted as shown in Table 1.

**Table 1:** Socio-demographic characteristics of the study participants at University of Gondar Comprehensive Specialized Hospital from April to September 2020 (n = 102).

Socio-demographic characteristics		Case		Control	
		Frequency (n)	%	Frequency(n)	%
**Age**	18–24	28	54.9	14	27.5
	25–34	19	37.3	26	50.9
	>35	4	7.8	11	21.6
**Marital status**	Single	40	78.4	21	41.2
	Married	10	19.6	24	47
	Divorced	1	2	5	9.8
	Widowed	0	0	1	2
**Residence**	Rural	40	78.4	11	21.6
	Urban	11	21.6	40	78.4
**Educational status**	Illiterate	40	78.4	4	7.5
	Primary school	8	15.7	11	21.6
	Secondary school	3	5.9	18	35.3
	College and University	0	0	18	35.3
**Occupation**	laborer	48	94.1	12	23.5
	Merchant	3	5.9	15	29.4
	Government employee	0	0	19	37.3
	Student	0	0	5	9.8
**Religion**	Orthodox	49	96.1	28	54.9
	Muslim	2	3.9	21	41.2
	Protestant	0	0	2	3.9

### Anthropometric measurements among the study participants

In this study, BMI of the study participants was calculated using their weight and height based on the formula BMI = weight/height^2^. The mean ± SD of BMI was found to be 16.1 ± 1.7 kg/m^2^ and 20.9 ± 2.5 kg/m^2^ for cases and control groups respectively. The mean value of BMI in VL patients failed under the category of underweight (<18.5) but the mean value of control groups was found within the normal category (18.5–24.9). The minimum and the maximum BMI values of patients were 13.5 kg/m^2^ and 19.7 kg/m^2^ respectively. On the other hand, the minimum and the maximum BMI values of control groups were 15.9 kg/m^2^ and 28.9 kg/m^2^ respectively. Based on WHO BMI classification, 46 (90.2%) and 5 (9.8%) of the patients failed under the category of underweight and normal weight respectively. In addition to this, there was no patient with overweight BMI value. On the other hand, 39 (76.5%), 9 (17.7%), and 3 (5.88%) of the control groups were normal weight, overweight, and underweight respectively. None of the study participants were obese as shown in Table 2.

**Table 2:** BMI categories of the study participants.

BMI	Case		Control	
	Frequency	%	Frequency	%
**Underweight (<18.5)**	46	90.2	9	17.7
**Normal weight (18.5–24.9)**	5	9.8	39	76.5
**Overweight (25–29.9)**	0	0	3	5.9
**Obese (≥ 30)**	0	0	0	0

### Comparison of liver function test levels among study participants

**Fig 1:**
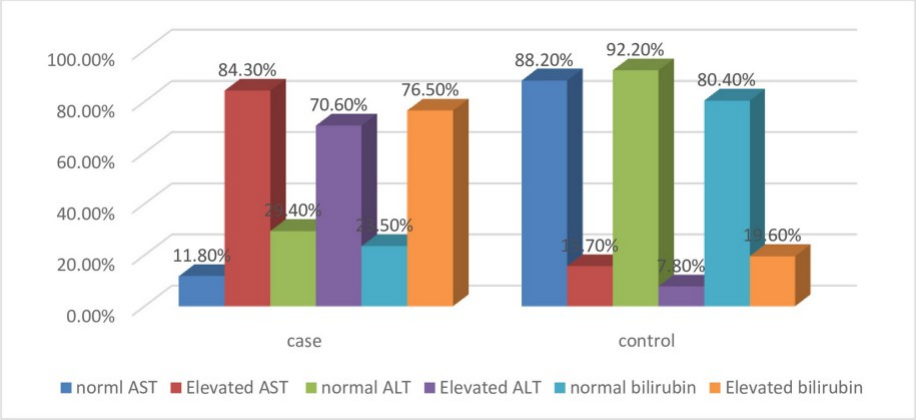
Categorical value of AST, ALT and total serum bilirubin among the study participants.

The mean ± SD of AST level in VL patients and apparently healthy controls were 61.8 ±20.4 and 23.8±10 respectively. Thus, the mean AST level showed a statistically significant elevation among cases as compared to controls (p<0.001) (Table 3). The mean ± SD of ALT level in VL patients and controls were 47.5 ±13.8 ± 28.2±10.5 respectively. The level of ALT was significantly higher among cases as compared to controls (P<0.001). Similarly, VL patients and control groups had a Mean ± SD value of 1.4±0.4 and 0.9±0.4 respectively. The total serum bilirubin value showed a statistically significant elevation among cases as compared to controls (P<0.001) (Table 3). The plasma level of AST was considered as normal if it falls within the ranges of 1-37U/L for adults. In this study, 43 (84.3%) of VL patients had values beyond the normal range (>37 U/L) and 8 (15.7%) of them had values within the normal range (1–37 U/L). From the apparently healthy controls, 45 (88.2%) and 6 (11.8%) of them had serum AST level within the normal and above the normal ranges respectively (Fig 1). Out of the total VL patients, 15 (29.4%) and 36 (70.6%) of them had normal (0–42 U/L) and elevated ALT level (above 42 U/L) respectively. From the total control group members, only 4 (7.8%) of them had elevated ALT level. However, 47 (92.2%) of them had the normal range of ALT value. Out of the total study participants, 12 (23.5%) VL patients and 41 (80.4%) control groups had the normal range (0–1.1 mg/dL) of total serum bilirubin value. However, 39 (76.5%) of cases and 10 (19.6%) of control groups had elevated total serum bilirubin level (> 1.1mg/dL) (Fig 1). The level of AST, ALT, and total bilirubin level of study participants were measured as shown below in Fig 1.

**Table 3:** Comparison of levels of AST, ALT, total serum bilirubin, serum albumin and total serum protein using independent sample t-test between VL patients and health controls.

Variables	Case (N = 51, mean± SD)	Control (N = 51, mean ±SD)	P-value
**AST**	61.8 ±20.4	23.8±10	<0.001*
**ALT**	47.5 ±13.8	28.2±10.5	<0.001*
**Total serum bilirubin**	1.4±0.4	0.9±0.4	<0.001*
**Serum albumin**	2.8 ±0.7	4.4 ±0.5	<0.001*
**Total protein**	5.9 ±1.2	7.4 ±0.7	<0.001*
**BMI**	16.1 ±1.6	20.9 ±2.5	<0.001*

(Independent t-test was done to compare mean ±SD of groups. Values are expressed as mean ± standard deviation.

(* = statistically significant at p-value <0.05).

### Comparison of nutritional status indicator biochemical parameters between VL patients and apparently healthy controls

Assessment of malnutrition status among VL patients and control groups was performed using anthropometric techniques and biochemical tests (serum albumin and total protein) through standard kit measurement method. There was a statistically significant reduction of serum albumin level in VL patients as compared to control groups (P< 0.001). Similarly, there was a statistically significant reduction of serum total protein in VL patients as compared to control groups (P< 0.001). Evaluation of the level of liver function tests and nutritional biochemical parameters are explained as shown below in the Table 3.

**Fig 2:**
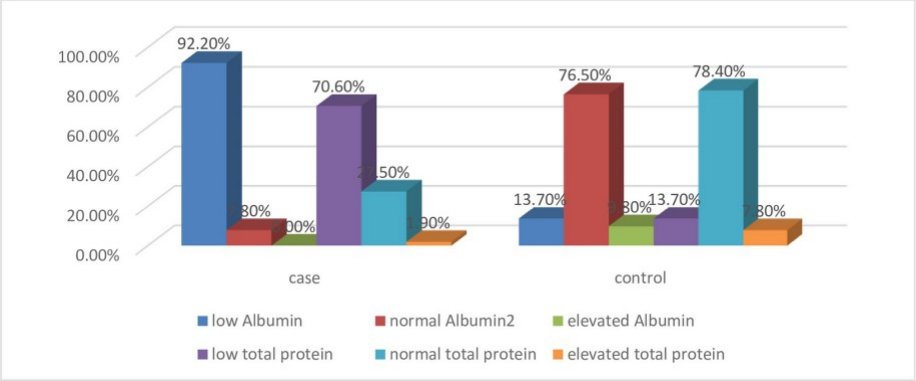
Evaluation of categorical value of serum albumin and serum total protein among the study participants.

In the other context, the level of serum albumin was considered as normal if it lies in between 3.8–5.1g/dL for adults. In this study, 47 (92.2%) and 4 (7.8%) of VL patients had below and within normal range of albumin level respectively. However, none of the patients had values above the normal range (Fig 2). From total enrolled controls, 7 (13.7%), 39 (76.5%), and 5 (9.8%) of them had normal range, within normal range, and above the normal range serum albumin level respectively (Fig 2). From the total study participants, 14 (27.5%) of VL patients and 40 (78.4%) of control groups had the normal range (6.6–8.7 g/dL) of serum total protein value. However, 36 (70.6%) of VL patients and 7 (13.7%) of controls showed below the normal value of serum total protein (>6.6mg/dL). On the other hand, 1 (1.9%) of patients and 4 (7.8%) of controls had elevated level of serum total protein (>8.7) (Fig 2). The level of serum total protein and albumin was evaluated as shown below in the Fig 2.

### Comparison of serum level of AST, ALT, total bilirubin, albumin and total protein with categorized age and BMI value

Serum level of AST, ALT, total bilirubin, albumin, and total protein were compared with different categories of age (18–24, 25–34 and ≥ 35) and BMI (underweight, normal weight, and overweight) using one way ANOVA analysis. The result showed that there was no a significant AST, bilirubin, and albumin difference in age category and BMI of cases and control groups. From the total enrolled patients, significant ALT mean differences was exhibited between 18–24 and 25–34 years old (P = 0.003 as shown in Tables 4 and 5). However, there was no significant mean ALT difference in BMI category. On the other hand, there was no mean difference in ALT level with age and BMI category among control groups. The serum total protein showed a statistically significant differences in the age category between 18–24 and ≥ 25–34 (P = 0.03 as shown in Tables 6 and 7) and between normal, and underweight among cases (p<0.001 as shown in Tables 8 and 9) respectively. In contrast, there was no a statistically significant total protein difference in age and BMI category among control groups. The comparison of serum level of AST, ALT, total bilirubin, albumin, and total protein with socio-demographic characteristics was evaluated as shown from Tables 4–9.

**Table 4:** Comparison of mean ALT with age categories among cases (one way ANOVA).

Analysis of Variance					
Source	SS	Df	MS	F	P-value
**Between groups**	2038.09926	2	1019.04963	6.59	**0.003**
**Within groups**	7420.5282	48	154.594337		
**Total**	9458.62745	50	189.172549		

**Table 5:** Pair-wise comparisons of ALT with categorized age using post hoc tests (Bonferroni).

Mean	18–24	25–34
**25–34**	-12.81	
	0.003*	
**> = 35**	-12.18	0.63
	0.219	1.000

(* = statistically significant at p-value <0.05).

**Table 6:** Comparison of mean total protein with age categories among cases (One way ANOVA).

Analysis of Variance					
Source	SS	Df	MS	F	P-value
**Between groups**	8.87001339	2	4.43500669	3.42	**0.0407**
**Within groups**	62.1674376	48	1.29515495		
**Total**	71.037451	50	1.42074902		

**Table 7:** Pair-wise comparisons of total protein with categorized age using post hoc tests (Bonferroni).

Mean	18–24	25–34
**25–34**	0.87	
	0.039*	
**> = 35**	0.60	-0.28
	0.990	1.000

(* = statistically significant at p-value <0.05).

**Table 8:** Comparison of mean total protein with BMI categories among cases (One way ANOVA).

Analysis of Variance					
Source	SS	Df	MS	F	P-value
**Between groups**	5.44392924	1	5.44392924	4.07	**0.0492**
**Within groups**	65.5935217	49	1.3386433		
**Total**	71.037451	50	1.42074902		

**Table 9:** Comparisons of total protein with categorized BMI using post hoc tests (Bonferroni).

Mean	Under Weight
**Normal Weight**	1.10
	0.049*

(* = statistically significant at p-value <0.05).

### Correlation analysis of socio-demographic profiles, anthropometric parameters with AST, ALT, total bilirubin, albumin and total protein of study participant

Bivariate Pearson correlation analysis showed that socio-demographic profile (age) showed a statistically significant and inverse correlation with AST (r = -0.3121, P = 0.0250) and ALT (r = -0.3394, P = 0.0148) value of VL patients. Body mass index (BMI) had a positive and statistically significant correlation with serum albumin level of VL patients (r = 0.2506 P = 0.050). Correlation analysis showed that the level of ALT was positively and significantly correlated with AST and total bilirubin level of VL patients as shown in the Table 10.

**Table 10:** Pearson’s correlation analysis between socio-demographic profiles, anthropometric parameters with AST, ALT, total bilirubin, albumin and total protein of study participant.

Variable			Age	BMI	ALT	Total protein
**AST**	Case	r-value	-0.3121	0.1005	0.3999	0.2181
		p-value	0.0250*	0.4829	0.0036*	0.1241
	Control	r-value	0.1162	-0.1595	0.5924	0.3810
		p-value	0.4169	0.2634	0.072	0.058
**ALT**	Case	r-value	-0.3394	-0.0814	1.000	0.2868
		p-value	0.0148*	0.5703	1.000	0.0413*
	Control	r-value	0.1637	-0.0335	1.000	0.0995
		p-value	0.2509	0.8155	1.000	0.4871
**Total bilirubin**	Case	r-value	-0.2233	0.1732		
		p-value	0.1152	0.2243		
	Control	r-value	0.2299	-0.1451		
		p-value	0.1046	0.3095		
**Albumin**	Case	r-value	0.0364	0.2506		
		p-value	0.7996	0.050*		
	Control	r-value	0.1078	-0.0183		
		p-value	0.4516	0.8988		
**Total protein**	Case	r-value	0.2199	0.1994		
		p-value	0.1210	0.1608		
	Control	r-value	-0.0485	-0.0734		
		p-value	0.7352	0.6086		

(r-value = pearson’s correlation coefficient,

* = statistically significant at P<0.05).

### Risk factors associated with ALT, AST, serum total bilirubin and serum total protein in VL patients

All variables with a p-value of less than 0.2 in the bivariable linear regression analysis were selected for multivariable linear regression analysis. All independent variables were checked by bivariate analysis. The result of the analysis showed that age, marital status, and residence of the VL participants had a significant association with serum AST level. However, the multivariate analysis showed that these factors did not significantly affect the level of AST. The bivariate analysis showed that age and educational status were significantly associated with ALT level of VL patients. Multivariate analysis showed that a one year increases in the age of cases decreases the serum AST by the factor of 0.78 by keeping other variables constant (Table 11). Age and BMI were significantly associated with total bilirubin during bivariate analysis, but both of them did not significantly affect the level of serum total bilirubin during multivariate analysis. All independent variables were checked by bivariate analysis, and indicate that age, residence occupation, educational status, religious belief, and BMI of the VL participants were found significantly associated with serum total protein. But, multivariate analysis showed that none of them significantly affected it.

**Table 11:** Multiple linear regression analysis of ALT, AST, serum total bilirubin and serum total protein in VL patients.

Variables	β (95% CI)	P-value
**AST**		
**Age**	-0.78	0.15
**Marital status**	9.03	0.20
**Residence**	-2.73	0.69
**ALT**		
**Age**	-0.78	0.02*
**Educational status**	-1.68	0.62
**Total Bilirubin**		
**Age**	-0.01	0.13
**BMI**	0.04	0.23
**Total Protein**		
**Age**	-0.28	0.33
**Residence**	0.29	0.52
**Occupation**	0.21	0.83
**Educational status**	0.48	0.23
**Religious belief**	-1.27	0.15
**BMI**	0.13	0.21

(β = Un Standardized coefficient,

* = statistically significant at P<0.05).

### Factors associated with ALT, AST, serum total bilirubin, and serum total protein

All independent variables of study participants were checked by bivariate analysis. Bivariate analysis showed that all predictor variables were significantly associated with biochemical profiles (ALT, AST, serum total bilirubin, and serum total protein). In multivariate regression analysis, being a patient was positively and significantly affects ALT, AST, and serum total bilirubin level and it also significantly and negatively affects serum total protein of study participant. Being VL patient increases the AST level of total study participants by 37.5 than controls. Being VL patient increases the ALT level of total study participants by 14.7 than controls. Additionally, it increases the total serum bilirubin level of total study participants by 0.68 than controls. In contrast to this, it also decreases the total protein level of total study participants by 1.01 than controls. In this study, age negatively and significantly affect the AST level of study participants. A one year increases in age decreases the AST level of total study participants by factor of 0.89 by keeping other variables constant (Table 12).

**Table 12:** Multiple linear regression analysis of factors associated with ALT, AST, serum total bilirubin and serum total protein.

	AST		ALT		Total bilirubin		Total protein	
Variable	β (95% CI)	P-value	β (95% CI)	P-value	β (95% CI)	P-value	β (95% CI)	P-value
**Status (44)**	37.5	0.00*	14.7	0.004*	0.68	0.00*	-1.01	0.02*
**Age**	-0.89	0.03*	-0.51	0.11	-0.01	0.51	0.001	0.98

(β = Un Standardized coefficient,

* = statistically significant at P<0.05).

## Discussion

Visceral leishmaniasis (VL) is a tropical disorder characterized by symptoms and laboratory tests resemble and misinterpreted with clinical characteristics of chronic liver disease. Patient with altered liver biochemical parameters often misleads the clinicians to diagnosis it as hepatitis. As a result, patient’s condition may worsen [12]. Previous clinical report on a patient with similar clinical and biochemical presentation to liver cirrhosis died with VL because the diagnosis took too long time [25]. As the parasite encountered with host immune system, it activates the immune response cascade, which in turn requires energy expenditure to be utilized by the body [8]. On the other hand, the parasites compute the nutrients with the host cell [26]. Consequently, it results in loss of nutrients and depletion of the reserved body nutrient. It also affects the metabolism and leads to malnutrition [8, 26].

From total enrolled 102 study participants, 51 of them were newly diagnosed VL patients and 51 of them were control groups. The Mean ± SD age of patients and controls were 24 ± 5.77 and 28.98 ± 6.43 years old respectively. Patients and controls had a minimum age of 18 and 19 years and a maximum age of 41 and 43 years respectively. In this study, all the study participants were male. Majority of VL patients (54.9%) were found within in the age group of 18–24 years and 50.9% of controls falls within the range of 25–34 years old. The result of this study was in line with a cross-sectional study done in Brazil, which included majority of young men with an average age of 28 years [27]. It may be due to most of our study participants with active VL were from endemic area, where labourers engaged with routine work activity that exposed to the parasite [28].

In this study, we estimated BMI value of the study participants by using the formula BMI = weight/height^2^. The Mean ± SD was found to be 16.1 ± 1.7 kg/m^2^ and 20.9 ± 2.5 kg/m^2^ for cases and control groups respectively. Thus, 90.2% of VL patients were underweight. Independent sample t-test was done, and it showed that there was a statistically significant difference in terms of BMI value among case and control groups (P<0.001). It was agreed with the study conducted in Brazil [29], which showed that BMI value was lowered for active VL patients (P < 0.0005). However, it was disagreed with the study done by Gatto M, et al., which showed that no significant differences in BMI value among patients and the control groups. Although there was no significant difference between patients and the control group, the pre-treatment patients had lower BMI values than the post-treatment patients and the controls [26]. The malnutrition associated with protein may be due to its low intake of nutrients as a result of severe infection that leads to losses of appetite, the host defense mechanism to induce depletion of energy [8, 26]. Furthermore, these variations may be associated with type of study participant, nutritional habits, and lifestyle difference among study participants.

In this study, AST and ALT were examined for patients and control groups. The study showed that a significant difference was exhibited in AST and ALT level among patients and controls. The serum level of ALT and AST was significantly higher among VL patients as compared to apparently healthy controls. Serum AST value was found significantly (p<0.001) higher among cases (61.8 ± 20.4) as compared to controls (23.8 ± 10). Serum ALT was also found significantly (p<0.001) higher among cases (47.5 ± 13.8) as compared to controls (28.2 ± 10.5). The result of this study was in line with previous reported similar studies, which confirmed a significant higher value of mean AST and ALT among VL patients. The serum AST level was found significantly (p<0.02) higher among cases (66 ± 54) as compared to controls (34 ± 11). Serum ALT was found significantly (p<0.05) higher among cases as compared to controls and the value of AST and ALT were higher (p-value <0.001 and 0.006) among cases respectively [11, 30].

The study conducted in Iraq also revealed that a highly significant increase (P < 0.05) in the level of ALT of VL patients was seen as compared to that of the mean value of apparently healthy control groups. The result of this study showed a highly significant increase (P < 0.05) in serum AST level of VL patients as compared to healthy control group, which was consistent with our study [31]. Furthermore, study done in India and case report in Italy supported the result of our study. Elevation of serum AST and ALT level as well as altered liver function were reported in those previous studies [12, 25]. This may be associated with parasite activated immune response in active VL, which results in initiation of the inflammatory processes and release of circulating cytokines. It may determine and lead to liver dysfunction and severe liver disease [9, 32]. Unlike our study, some studies showed that no significant difference among case and control, which disagreed with our result [33, 34]. Study conducted in Iraq showed that the mean value of ALT and AST were under the normal range [35]. Sample size, study design, geographical area, genetic makeup, reference value of clinical chemistry tests, and lifestyle differences among study participants may contribute to this variation.

The mean total serum bilirubin was significantly higher (P<0.001) among cases (1.4 ± 0.4) as compared to controls (0.9 ± 0.4). The result of this study was in line with the study conducted in Sudan and Iraq [12, 25], which showed that serum bilirubin was significantly higher in VL patients than healthy controls. Similarly, case report conducted in Italy revealed that elevated total bilirubin exhibited in VL patients [12, 25, 36]. This may be due to a consequence of parasitic infiltration in the major organ, including liver, bone marrow, and spleen. Therefore, it increases the size of these organs and decrease size of blood cells, which makes the red blood cells prone to hemolysis, sequestration and destruction [10]. Our result was disagreed with the study done in Iraq, which showed that no significant difference (p = 0.395) in the mean value of serum bilirubin among VL patients and controls [37]. This variation might be due to differences in study participant, geographical area, genetic makeup, reference value of clinical chemistry analyzers, and lifestyle.

On the other hand, serum albumin and total protein were examined for patients and apparently healthy control groups. The result of this study showed that there was a statistically significant difference in the level of serum albumin and total protein. There was a statistically significant (p< 0.001) reduction of serum albumin in VL patients as compared to control groups. Our results were similar to different studies done in India, Brazil, and Sudan [30, 38, 39], which showed that serum albumin was significantly lowered (p<0.001) among VL patients as compared to controls. Besides, study done in brazil also reported that pre-treatment patients had lower albumin levels as compared to post-treatment and the healthy control groups (p-value <0.05) [40]. Furthermore, study done in Brazil and Iraq also supported our finding and showed that serum albumin level was low as compared to the previous studies [33, 35]. It may be attributed to protein energy malnutrition either due to its low intake of nutrients and severe infection of VL that leads to losses of appetite or the host defense mechanism to induce depletion of energy [40].

In our study, serum total protein level showed a statistically significant (P< 0.001) reduction in VL patients as compared to control groups. It was in contrast to other studies done in India and Brazil [41]. These studies showed that there was no statistically significant difference in total protein levels among cases and control groups. On the other hand, another study done in Brazil reported that the total protein was increased in the patient as compared to the normal values and the variation was statistically significant for the control groups (p < 0.05) [33]. Our finding of lowered total protein may be associated with low serum albumin and low BMI, which in turn associated with a decrease in serum protein levels [42]. The variation may be due to a difference in nutritional habits, BMI, reference value of chemistry analyzer, lifestyle, and study participant [33, 39].

The age was significantly and inversely correlated with AST value of VL patients, but other variables did not correlate with the dependent variables. This finding was in line with study done in Brazil [27], which reported that liver function test derangement among young VL patients. The result of this study was contradicted with the study done in Brazil [43], which showed that age increased the likelihood of having altered liver function. Furthermore, report done on effects of aging on the liver function [41], which showed that age was significantly and positively correlate with liver function tests. This variation may be due to a difference in age group of study participants, study design, severity of the disease, and interaction of the VL patients with parasites.

Body mass index (BMI) was positively and significantly correlated with serum albumin. As BMI decreases the level of serum albumin also decreases. However, there was no significant association with AST, total bilirubin, and total protein level of VL patients (P = 0.050). During our study period, there were no any scientific data that deals about the association of BMI with liver function tests and total protein. The finding may be associated with protein energy malnutrition that leads to a decrease in albumin and BMI of VL patients [40]. In addition, our study showed that there was a correlation between ALT, AST, and total bilirubin. The correlation analysis of this study showed that the level of ALT was positively and significantly correlated with AST and total bilirubin level of VL patients. Similarly, there were no previous studies done regarding their correlation up to our scope of finding literature. In this study, AST, ALT, bilirubin, and total protein alteration has been demonstrated in VL patients. Being VL patient positively and significantly affects ALT, AST, and serum total bilirubin, and it also significantly and negatively affects serum total protein of study participant. Visceral leshimaniasis (VL) was also associated with an elevation of AST, ALT, bilirubin, and reduction in total protein as compared to healthy control group.

## Conclusion

The result of this study showed a statistically significant elevation in mean values of serum AST, ALT, and total bilirubin level. Lower mean level of albumin and total protein was identified among VL patients as compared to controls. The significant change in serum AST, ALT, and total bilirubin level was associated with an abnormality in normal function of liver, which in turn caused by leishmania parasite-mediated immune response. The marked change and derangement in liver function biochemical profiles, including serum albumin and total protein showed the effect VL associated complication. Consequently, VL was associated with marked elevation of AST, ALT, bilirubin, and reduction in total protein as compared to healthy control group. Socio-demographic profile (age) of the participant negatively and significantly affect the AST level of study participants. Therefore, patients with different clinical stage of this disease showed a change in biochemical and nutritional parameters, including liver enzymes, hyperbilirubinemia, hypoalbuminemia, and hypoproteinemia. Generally, VL patients showed statistically significant alteration of liver and nutritional status indicator biochemical parameters as compared to controls. Consequently, VL leads to liver dysfunction and malnutrition condition.

## List of abbreviation

ALT: Alanine amino transferase
AST: Aspartate amino transferase
BMI: Body Mass Index
VL: Visceral leishmaniasis
